# Supplementary material for: Burden of Illness beyond Mortality and Heart Failure Hospitalizations in Patients Newly Diagnosed with Heart Failure in Spain According to Ejection Fraction
Source: J Clin Med. 2023 Mar 21;12(6):2410. doi: 10.3390/jcm12062410 (PMC10054096; doi:10.3390/jcm12062410)
Supplement: Supplementary file 1 [file jcm-12-02410-s001.zip › jcm-2209374-supplementary.pdf]

Supplementary Figure S1. Survival free of adverse clinical outcomes.

1.a. Survival free of the composite MACE Outcome: overall and by EF subgroups.

Overall

Time (months)	**0**	**6**	**12**	**18**	**24**	**30**	**36**
No at risk	19961	19818	19338	17111	14381	11909	9812
Cum no of events	0	598	2155	3247	4303	5312	5942

HFrEF

Time (months)	**0**	**6**	**12**	**18**	**24**	**30**	**36**
No at risk	8678	8615	8394	7431	6242	5223	4348
Cum no of events	0	314	1127	1655	2164	2636	2898

HFpEF

Time (months)	**0**	**6**	**12**	**18**	**24**	**30**	**36**
No at risk	5244	5202	5076	4471	3760	3137	2600
Cum no of events	0	137	469	730	978	1220	1385

HFmrEF

Time (months)	**0**	**6**	**12**	**18**	**24**	**30**	**36**
No at risk	1022	1016	990	907	760	609	476
Cum no of events	0	26	99	145	200	248	290

HFuEF

Time (months)	**0**	**6**	**12**	**18**	**24**	**30**	**36**
No at risk	5017	4985	4878	4302	3619	2940	2388
Cum no of events	0	121	460	718	963	1210	1371

1.b. Survival free of stroke: overall and by EF subgroups.

Overall

Time (months)	**0**	**6**	**12**	**18**	**24**	**30**	**36**
No at risk	19961	19818	19338	17111	14381	11909	9812
Cum no of events	0	190	698	924	1044	1149	1195

HFrEF

Time (months)	**0**	**6**	**12**	**18**	**24**	**30**	**36**
No at risk	8678	8615	8394	7431	6242	5223	4348
Cum no of events	0	88	363	486	555	618	652

HFpEF

Time (months)	**0**	**6**	**12**	**18**	**24**	**30**	**36**
No at risk	5244	5202	5076	4471	3760	3137	2600
Cum no of events	0	43	152	196	214	233	237

HFmrEF

Time (months)	**0**	**6**	**12**	**18**	**24**	**30**	**36**
No at risk	1022	1016	990	907	760	609	476
Cum no of events	0	12	29	36	42	45	48

HFuEF

Time (months)	**0**	**6**	**12**	**18**	**24**	**30**	**36**
No at risk	5017	4985	4878	4302	3619	2940	2388
Cum no of events	0	47	154	206	233	253	258

1.c. Survival free of all-cause mortality: overall and by EF subgroups.

Overall

Time (months)	0	6	12	18	24	30	36
No at risk	19961	19818	19338	17111	14381	11909	9812
Cum no of events	0	281	906	1532	2391	3195	3770

HFrEF

Time (months)	0	6	12	18	24	30	36
No at risk	8678	8615	8394	7431	6242	5223	4348
Cum no of events	0	169	490	878	1382	1846	2157

HFpEF

Time (months)	0	6	12	18	24	30	36
No at risk	5244	5202	5076	4471	3760	3137	2600
Cum no of events	0	57	211	317	471	628	733

HFmrEF

Time (months)	0	6	12	18	24	30	36
No at risk	1022	1016	990	907	760	609	476
Cum no of events	0	8	32	50	86	115	152

HFuEF

Time (months)	0	6	12	18	24	30	36
No at risk	5017	4985	4878	4302	3619	2940	2388
Cum no of events	0	47	173	287	452	606	728

1.d. Survival free of myocardial infarction: overall and by EF subgroups.

Overall

Time (months)	0	6	12	18	24	30	36
No at risk	19961	19818	19338	17111	14381	11909	9812
Cum no of events	0	254	924	1281	1434	1574	1639

HFrEF

Time (months)	0	6	12	18	24	30	36
No at risk	8678	8615	8394	7431	6242	5223	4348
Cum no of events	0	159	536	702	781	860	893

HFpEF

Time (months)	0	6	12	18	24	30	36
No at risk	5244	5202	5076	4471	3760	3137	2600
Cum no of events	0	50	172	255	294	319	331

HFmrEF

Time (months)	0	6	12	18	24	30	36
No at risk	1022	1016	990	907	760	609	476
Cum no of events	0	7	40	61	69	75	78

HFuEF

Time (months)	0	6	12	18	24	30	36
No at risk	5017	4985	4878	4302	3619	2940	2388
Cum no of events	0	38	176	263	290	320	337

1.e . Survival free of HF hospitalization: overall and by EF subgroups.

Overall

Time (months)	**0**	**6**	**12**	**18**	**24**	**30**	**36**
No at risk	19961	19818	19338	17111	14381	11909	9812
Cum no of events	0	962	2384	4160	5183	5986	6491

HFrEF

Time (months)	**0**	**6**	**12**	**18**	**24**	**30**	**36**
No at risk	8678	8615	8394	7431	6242	5223	4348
Cum no of events	0	649	1471	2278	2680	3068	3324

HFpEF

Time (months)	**0**	**6**	**12**	**18**	**24**	**30**	**36**
No at risk	5244	5202	5076	4471	3760	3137	2600
Cum no of events	0	122	380	819	1073	1258	1372

HFmrEF

Time (months)	**0**	**6**	**12**	**18**	**24**	**30**	**36**
No at risk	1022	1016	990	907	760	609	476
Cum no of events	0	49	112	204	273	322	347

HFuEF

Time (months)	**0**	**6**	**12**	**18**	**24**	**30**	**36**
No at risk	5017	4985	4878	4302	3619	2940	2388
Cum no of events	0	142	421	859	1157	1338	1448

1.f. Survival free of HF hospitalization and all-cause mortality: overall and by EF subgroups.

Overall

Time (months)	**0**	**6**	**12**	**18**	**24**	**30**	**36**
No at risk	19961	19818	19338	17111	14381	11909	9812
Cum no of events	0	1058	2742	4880	6458	7826	8718

HFrEF

Time (months)	**0**	**6**	**12**	**18**	**24**	**30**	**36**
No at risk	8678	8615	8394	7431	6242	5223	4348
Cum no of events	0	684	1619	2591	3244	3865	4265

HFpEF

Time (months)	**0**	**6**	**12**	**18**	**24**	**30**	**36**
No at risk	5244	5202	5076	4471	3760	3137	2600
Cum no of events	0	155	488	1024	1419	1769	1998

HFmrEF

Time (months)	**0**	**6**	**12**	**18**	**24**	**30**	**36**
No at risk	1022	1016	990	907	760	609	476
Cum no of events	0	52	130	240	334	406	455

HFuEF

Time (months)	**0**	**6**	**12**	**18**	**24**	**30**	**36**
No at risk	5017	4985	4878	4302	3619	2940	2388
Cum no of events	0	167	505	1025	1461	1786	2000

Abbreviations: EF = Ejection fraction; HF = Heart failure; HFmrEF = Heart failure with mildly reduced ejection fraction; HFpEF = Heart Failure with preserved ejection fraction; HFrEF = Heart failure with reduced ejection fraction; HFuEF = Heart Failure with unspecified ejection fraction; MACE = Major adverse cardiovascular events.

Supplementary Table S1. Evolution of HF treatment in the overall incident HF Cohort (Index Date 2013 – 2019; n=19961)

	Baseline (n=19961)	12 months (n=19309)	P
Diuretics, n (%)	13845 (69.4)	14249 (73.8)	<0.01
Beta-blockers	13992 (70.1)	13965 (72.3)	<0.01
RAASi	11245 (56.3)	12611 (65.4)	<0.01
ACEi/ARB	10026 (50.2)	10455 (54.2)	<0.01
ARNI	1219 (6.1)	2156 (11.2)	<0.01
MRA	2360 (11.8)	3655 (18.9)	<0.01
SGLT2i	1779 (8.9)	1895 (9.8)	0.002
Digoxin	4007 (20.1)	4481 (23.2)	<0.01
Ivabradine	1218 (6.1)	1471 (7.6)	<0.01
Hydralazine and nitrate	14 (0.07)	135 (0.7)	<0.01

RAASi: renin angiotensin system inhibitors; ACEi: angiotensin-converting enzyme inhibitors; ARB: angiotensin receptor blockers; ARNI: angiotensin II receptor antagonist and a neprilysin inhibitor; MRA: mineralocorticoid receptor antagonists.

Supplementary Table S2. Incidence Rate of MACE Outcomes in the Incident HF Cohort by Years since Index Date

	All HF (N=19961)	HFrEF (N=8678)	HFmrEF (N=1022)	HFpEF (N=5244)	HFpEF (50 to <60%) (N=1833)	HFpEF (≥60%) (N=3411)	HFuEF (N=5017)
**1 Year Since Index Date**							
**Myocardial infarction**							
Patients with outcome (n)	941	543	40	178	79	99	180
Total person-years	19412	8375	1000	5124	1786	3338	4912
Rate per 1,000 person-years (95% CI)	48.5 (45.5 - 51.6)	64.8 (59.8 - 70.3)	40 (29.5 - 54)	34.7 (30.1 - 40.1)	44.2 (35.6 - 54.8)	29.7 (24.4 - 36)	36.6 (31.7 - 42.3)
**Stroke**							
Patients with outcome (n)	710	368	30	154	62	92	158
Total person-years	19516	8462	1001	5137	1791	3345	4916
Rate per 1,000 person-years (95% CI)	36.4 (33.8 - 39.1)	43.5 (39.3 - 48)	30 (21.1 - 42.4)	30 (25.7 - 35)	34.6 (27.1 - 44.1)	27.5 (22.5 - 33.6)	32.1 (27.6 - 37.4)
**All-cause mortality**							
Patients with outcome (n)	650	298	30	177	61	116	145
Total person-years	19729	8568	1012	5184	1813	3372	4964
Rate per 1,000 person-years (95% CI)	32.9 (30.5 - 35.5)	34.8 (31.1 - 38.9)	29.6 (20.8 - 42)	34.1 (29.5 - 39.4)	33.6 (26.3 - 43)	34.4 (28.8 - 41.1)	29.2 (24.9 - 34.3)
**Composite MACE**		1					
Patients with outcome (n)	2234	158	100	494	193	301	482
Total person-years	19167	6109	988	5071	1764	3307	4853
Rate per 1,000 person-years (95% CI)	116.6 (112.1 - 121.2)	189.6 (179.9 - 199.6)	101.2 (83.9 - 121.6)	97.4 (89.6 - 105.9)	109.4 (95.7 - 124.8)	91 (81.7 - 101.3)	99.3 (91.2 - 108.1)
**HF: hospitalization**							
Patients with outcome (n)	2422	1494	115	383	149	234	430
Total person-years	18.731	7.906	964	5.046	1.758	3.288	4.815
Rate per 1,000 person-years (95% CI)	129.3 (124.6 - 134.2)	189 (180,5 - 197,7)	119.3 (100.3 - 141.3)	75.9 (68.9 - 83.5)	84.7 (72.6 - 98.7)	71.2 (62.9 - 80.5)	89.3 (81.6 - 97.7)
**HF: hospitalization and mortality**							
Patients with outcome (n)	2799	1649	133	499	184	315	518
Total person-years	18.731	7.906	964	5.046	1.758	3.288	4.815
Rate per 1,000 person-years (95% CI)	149.4 (144.4 - 154.6)	208.6 (199.8 - 217.7)	138 (117.6 - 161.2)	98.9 (91 - 107.4)	104.6 (91.2 - 119.8)	95.8 (86.2 - 106.3)	107.6 (99.1 - 116.7)
**2 Years Since Index Date**							
**Myocardial infarction**							
Patients with outcome (n)	496	240	29	116	42	74	111
Total person-years	15838	6741	844	4208	1463	2744	4046
Rate per 1,000 person-years (95% CI)	31.3 (28.7 - 34.1)	35.6 (31.4 - 40.3)	34.4 (24 - 48.9)	27.6 (23 - 33)	28.7 (21.3 - 38.6)	27 (21.5 - 33.7)	27.4 (22.8 - 32.9)
**Stroke**							
Patients with outcome (n)	342	189	12	62	25	37	79
Total person-years	16209	6971	859	4275	1502	2772	4105
Rate per 1,000 person-years (95% CI)	21.1 (19 - 23.4)	27.1 (23.6 - 31.2)	14 (8 - 24.3)	14.5 (11.3 - 18.5)	16.6 (11.3 - 24.5)	13.3 (9.7 - 18.3)	19.2 (15.5 - 23.9)
**All-cause mortality**							
Patients with outcome (n)	1671	834	75	383	148	235	379
Total person-years	16789	7264	892	4402	1547	2855	4231
Rate per 1,000 person-years (95% CI)	99.5 (95.1 - 104.2)	114.8 (107.7 - 122.3)	84.1 (67.6 - 104.2)	87 (79 - 95.7)	95.7 (82 - 111.4)	82.3 (72.8 - 93)	89.6 (81.3 - 98.6)
**Composite MACE**							
Patients with outcome (n)	2181	1070	103	502	189	313	506
Total person-years	15145	6393	811	4058	1411	2647	3883
Rate per 1,000 person-years (95% CI)	144 (138.5 - 149.7)	167.4 (158.4 - 176.7)	127 (105.8 - 151.7)	123.7 (113.9 - 134.2)	134 (117.2 - 152.7)	118.2 (106.5 - 131.1)	130.3 (120.1 - 141.3)
**HF: hospitalization**							
Patients with outcome (n)	2804	1202	163	704	249	455	735

Total person-years	13.931	5.673	735	3.874	1.359	2.515	3.648
Rate per 1,000 person-years (95% CI)	201.3 (194.7 - 208)	211.9 (201.4 - 222.7)	221.7 (193.2 - 253.2)	181.7 (169.9 - 194.2)	183.2 (163.6 - 204.7)	180.9 (166.3 - 196.4)	201.5 (188.8 - 214.8)
**HF: hospitalization and mortality**							
Patients with outcome (n)	3733	1627	207	940	335	605	959
Total person-years	13.931	5.673	735	3.874	1.359	2.515	3.648
Rate per 1,000 person-years (95% CI)	268 (260.7 - 275.4)	286.8 (275.2 - 298.7)	281.6 (250.3 - 315.2)	242.6 (229.4 - 256.4)	246.5 (224.3 - 270.1)	240.5 (224.2 - 257.6)	262.9 (248.9 - 277.4)
**3 Years Since Index Date**							
**Myocardial infarction**							
Patients with outcome (n)	207	112	9	39	14	25	47
Total person-years	10895	4683	561	2916	1010	1906	2734
Rate per 1,000 person-years (95% CI)	19 (16.6 - 21.7)	23.9 (19.9 - 28.7)	16 (8.5 - 30.2)	13.4 (9.8 - 18.2)	13.9 (8.3 - 23.1)	13.1 (8.9 - 19.3)	17.2 (13 - 22.8)
**Stroke**							
Patients with outcome (n)	149	100	6	21	11	10	22
Total person-years	11225	4890	568	2989	1045	1944	2778
Rate per 1,000 person-years (95% CI)	13.3 (11.3 - 15.6)	20.4 (16.8 - 24.8)	10.6 (4.9 - 22.9)	7 (4.6 - 10.7)	10.5 (5.9 - 18.7)	5.1 (2.8 - 9.4)	7.9 (5.2 - 12)
**All-cause mortality**							
Patients with outcome (n)	1768	821	93	433	169	264	421
Total person-years	11786	5135	605	3125	1093	2032	2920
Rate per 1,000 person-years (95% CI)	150 (143.7 - 156.6)	159.9 (150.1 - 170.1)	153.7 (127.1 - 184.6)	138.6 (126.9 - 151.1)	154.7 (134.5 - 177.3)	129.9 (116 - 145.2)	144.2 (131.9 - 157.4)
**Composite MACE**							
Patients with outcome (n)	1620	731	91	400	149	251	398
Total person-years	10438	4435	539	2832	983	1850	2632
Rate per 1,000 person-years (95% CI)	155.2 (148.4 - 162.3)	164.8 (154.2 - 176)	168.9 (139.6 - 202.8)	141.2 (128.9 - 154.5)	151.6 (130.6 - 175.4)	135.7 (120.8 - 152.1)	151.2 (138 - 165.4)
**HF: hospitalization**							
Patients with outcome (n)	1313	648	71	296	106	190	298
Total person-years	8.813	3.621	437	2.493	888	1.605	2.262
Rate per 1,000 person-years (95% CI)	149 (141.7 - 156.6)	179 (166.8 - 191.8)	162.6 (131 - 200.2)	118.8 (106.6 - 132)	119.4 (99.7 - 142.4)	118.4 (103.5 - 135.1)	131.7 (118.4 - 146.3)
**HF: hospitalization and mortality**							
Patients with outcome (n)	2262	1021	118	577	216	361	546
Total person-years	8.813	3.621	437	2.493	888	1.605	2.262
Rate per 1,000 person-years (95% CI)	256.7 (247.7 - 265.9)	282 (267.5 - 296.8)	270.3 (230.8 - 313.8)	231.5 (215.3 - 248.4)	243.3 (216.2 - 272.6)	224.9 (205.2 - 246)	241.3 (224.2 - 259.4)
**4 Years Since Index Date**							
**Myocardial infarction**							
Patients with outcome (n)	74	47	3	10	2	8	14
Total person-years	7.38	3183	350	1985	676	1309	1820
Rate per 1,000 person-years (95% CI)	10.1 (8 - 12.6)	14.8 (11.1 - 19.6)	8.6 (2.9 - 24.9)	5 (2.7 - 9.2)	3 (0.8 - 10.7)	6.1 (3.1 - 12)	7.7 (4.6 - 12.9)
**Stroke**							
Patients with outcome (n)	99	70	2	9	5	4	18
Total person-years	7576	3318	355	2057	705	1352	1845
Rate per 1,000 person-years (95% CI)	13.1 (10.7 - 15.9)	21.1 (16.7 - 26.6)	5.6 (1.5 - 20.3)	4.4 (2.3 - 8.3)	7.1 (3 - 16.5)	3 (1.2 - 7.6)	9.8 (6.2 - 15.4)
**All-cause mortality**							
Patients with outcome (n)	1033	543	47	223	72	151	220
Total person-years	7912	3463	376	2136	733	1403	1937
Rate per 1,000 person-years (95% CI)	130.6 (123.3 - 138.2)	156.8 (145.1 - 169.3)	125.1 (95.4 - 162.4)	104.4 (92.1 - 118.1)	98.3 (78.8 - 122)	107.6 (92.4 - 124.9)	113.6 (100.2 - 128.5)
**Composite MACE**							
Patients with outcome (n)	860	447	39	190	67	123	184
Total person-years	6937	2967	328	1915	649	1266	1727
Rate per 1,000 person-years (95% CI)	124 (116.4 - 131.9)	150.6 (138.2 - 164)	118.8 (88.1 - 158.3)	99.2 (86.6 - 113.4)	103.3 (82.1 - 129.1)	97.2 (82 - 114.7)	106.6 (92.9 - 122)

**HF: hospitalization**							
Patients with outcome (n)	533	205	31	151	52	99	146
Total person-years	5.529	2.294	253	1.585	550	1.035	1.398
Rate per 1,000 person-years (95% CI)	96.4 (88.9 - 104.5)	89.4 (78.4 - 101.7)	122.7 (87.8 - 169)	95.3 (81.8 - 110.7)	94.5 (72.8 - 121.9)	95.7 (79.2 - 115.1)	104.4 (89.5 - 121.6)
**HF: hospitalization and mortality**							
Patients with outcome (n)	1075	477	58	282	97	185	258
Total person-years	5.529	2.294	253	1.585	550	1.035	1.398
Rate per 1,000 person-years (95% CI)	194.4 (184.2 - 205.1)	207.9 (191.8 - 225)	229.6 (182.1 - 285.3)	177.9 (159.9 - 197.5)	176.4 (146.8 - 210.4)	178.8 (156.6 - 203.3)	184.6 (165.1 - 205.7)
**5 Years Since Index Date**							
**Myocardial infarction**							
Patients with outcome (n)	66	40	3	16	7	9	7
Total person-years	4.72	2042	220	1296	432	863	1115
Rate per 1,000 person-years (95% CI)	14.1 (11.1 - 17.9)	19.6 (14.4 - 26.6)	13.6 (4.6 - 39.3)	12.3 (7.6 - 20)	16.2 (7.9 - 33)	10.4 (5.5 - 19.7)	6.3 (3 - 12.9)
**Stroke**							
Patients with outcome (n)	56	41	2	4	1	3	9
Total person-years	4802	2108	227	1347	457	890	1119
Rate per 1,000 person-years (95% CI)	11.7 (9 - 15.1)	19.5 (14.4 - 26.3)	8.8 (2.4 - 31.5)	3 (1.2 - 7.6)	2.2 (0.4 - 12.3)	3.4 (1.1 - 9.9)	8 (4.2 - 15.2)
**All-cause mortality**							
Patients with outcome (n)	669	375	24	131	46	85	139
Total person-years	5102	2245	242	1416	481	935	1198
Rate per 1,000 person-years (95% CI)	131.1 (122.1 - 140.7)	167 (152.1 - 183)	99 (67.4 - 143)	92.5 (78.5 - 108.7)	95.6 (72.5 - 125.2)	90.9 (74.1 - 111.1)	116 (99.1 - 135.4)
**Composite MACE**							
Patients with outcome (n)	632	338	26	134	46	88	134
Total person-years	3576	1871	207	1238	409	828	1041
Rate per 1,000 person-years (95% CI)	176.8 (164.6 - 189.6)	180.7 (163.9 - 198.8)	125.7 (87.2 - 177.8)	108.3 (92.1 - 126.8)	112.4 (85.3 - 146.7)	106.2 (87 - 129.1)	128.7 (109.7 - 150.4)
**HF: hospitalization**							
Patients with outcome (n)	253	147	13	45	17	28	48
Total person-years	3.329	1.403	146	975	332	642	805
Rate per 1,000 person-years (95% CI)	76 (67.5 - 85.5)	104.8 (89.8 - 121.9)	89.2 (52.9 - 146.6)	46.2 (34.7 - 61.2)	51.2 (32.2 - 80.4)	43.6 (30.3 - 62.3)	59.6 (45.2 - 78.1)
**HF: hospitalization and mortality**							
Patients with outcome (n)	626	343	25	129	45	84	129
Total person-years	3.329	1.403	146	975	332	642	805
Rate per 1,000 person-years (95% CI)	188 (175.1 - 201.7)	244.5 (222.7 - 267.6)	171.5 (119 - 241)	132.3 (112.5 - 155.1)	135.4 (102.8 - 176.4)	130.7 (106.9 - 159)	160.2 (136.4 - 187.1)
**6 Years Since Index Date**							
**Myocardial infarction**							
Patients with outcome (n)	38	24	1	8	3	5	5
Total person-years	2719	1213	122	734	243	491	649
Rate per 1,000 person-years (95% CI)	14 (10.2 - 19.1)	19.8 (13.3 - 29.3)	8.2 (1.4 - 45)	10.9 (5.5 - 21.4)	12.3 (4.2 - 35.7)	10.2 (4.4 - 23.6)	7.7 (3.3 - 17.9)
**Stroke**							
Patients with outcome (n)	37	32	0	2	1	1	3
Total person-years	2781	1236	127	774	263	511	644
Rate per 1,000 person-years (95% CI)	13.3 (9.7 - 18.3)	25.9 (18.4 - 36.3)	0 (0 - 29.3)	2.6 (0.7 - 9.4)	3.8 (0.7 - 21.2)	2 (0.3 - 11)	4.7 (1.6 - 13.6)
**All-cause mortality**							
Patients with outcome (n)	460	340	14	48	19	29	58
Total person-years	3031	1364	140	824	281	543	703
Rate per 1,000 person-years (95% CI)	151.8 (139.4 - 165)	249.2 (227 - 272.8)	100.2 (60.6 - 161.2)	58.2 (44.2 - 76.4)	67.7 (43.7 - 103.3)	53.4 (37.4 - 75.6)	82.5 (64.4 - 105.2)
**Composite MACE**							
Patients with outcome (n)	342	284	6	27	12	15	25

Total person-years	2479	1076	112	695	227	468	596
Rate per 1,000 person-years (95% CI)	138 (125 - 152.1)	264 (238.5 - 291.1)	53.8 (24.9 - 112.4)	38.9 (26.8 - 55.9)	53 (30.5 - 90.3)	32 (19.5 - 52.2)	41.9 (28.6 - 61.1)
**HF: hospitalization**							
Patients with outcome (n)	93	42	3	18	6	12	30
Total person-years	1.863	799	81	541	188	353	443
Rate per 1,000 person-years (95% CI)	49.9 (40.9 - 60.8)	52.6 (39.1 - 70.3)	37.1 (12.7 - 103.4)	33.3 (21.2 - 52)	32 (14.7 - 68)	34 (19.6 - 58.5)	67.8 (47.9 - 95.1)
**HF: hospitalization and mortality**							
Patients with outcome (n)	317	229	7	34	14	20	47
Total person-years	1.863	799	81	541	188	353	443
Rate per 1,000 person-years (95% CI)	170.1 (153.8 - 187.9)	286.6 (256.3 - 318.9)	86.5 (42.5 - 167.9)	62.9 (45.4 - 86.6)	74.6 (45 - 121.3)	56.7 (37 - 85.9)	106.2 (80.8 - 138.3)
**7 Years Since Index Date**							
**Myocardial infarction**							
Patients with outcome (n)	26	20	0	3	1	2	3
Total person-years	1231	561	47	339	115	223	285
Rate per 1,000 person-years (95% CI)	21.1 (14.5 - 30.8)	35.7 (23.2 - 54.5)	0 (0 - 75)	8.9 (3 - 25.7)	8.7 (1.5 - 47.6)	9 (2.5 - 32)	10.5 (3.6 - 30.5)
**Stroke**							
Patients with outcome (n)	19	14	0	1	0	1	4
Total person-years	1232	543	50	355	122	233	285
Rate per 1,000 person-years (95% CI)	15.4 (9.9 - 24)	25.8 (15.4 - 42.8)	0 (0 - 70.9)	2.8 (0.5 - 15.8)	0 (0 - 30.5)	4.3 (0.8 - 23.9)	14 (5.5 - 35.6)
**All-cause mortality**							
Patients with outcome (n)	303	229	6	36	18	18	32
Total person-years	1396	641	57	382	131	251	316
Rate per 1,000 person-years (95% CI)	217.1 (196.2 - 239.5)	357.2 (321.1 - 395.1)	104.5 (48.8 - 209.8)	94.3 (68.9 - 127.8)	137.9 (89.1 - 207.5)	71.6 (45.8 - 110.3)	101.4 (72.8 - 139.7)
**Composite MACE**							
Patients with outcome (n)	169	148	0	4	1	3	17
Total person-years	1094	470	42	321	107	214	260
Rate per 1,000 person-years (95% CI)	154.5 (134.3 - 177.2)	314.7 (274.4 - 358.1)	0 (0 - 83.2)	12.5 (4.9 - 31.6)	9.3 (1.6 - 50.9)	14 (4.8 - 40.4)	65.4 (41.2 - 102.2)
**HF: hospitalization**							
Patients with outcome (n)	38	17	3	7	4	3	11
Total person-years	807	364	33	234	83	151	177
Rate per 1,000 person-years (95% CI)	47.1 (34.5 - 64)	46.7 (29.4 - 73.6)	92 (31.8 - 238.3)	29.9 (14.6 - 60.5)	48.3 (18.9 - 117.7)	19.9 (6.8 - 56.8)	62.2 (35.1 - 107.9)
**HF: hospitalization and mortality**							
Patients with outcome (n)	138	108	3	7	4	3	20
Total person-years	807	364	33	234	83	151	177
Rate per 1,000 person-years (95% CI)	171 (146.6 - 198.6)	296.9 (252.3 - 345.8)	92 (31.8 - 238.3)	29.9 (14.6 - 60.5)	48.3 (18.9 - 117.7)	19.9 (6.8 - 56.8)	113.1 (74.4 - 168.2)

Abbreviations: CI = Confidence interval; HF = Heart failure; HFmrEF = Heart failure with mildly reduced ejection fraction; HFpEF = Heart Failure with preserved ejection fraction; HFrEF = Heart failure with reduced ejection fraction; HFuEF = Heart Failure with unspecified ejection fraction; HR: Hazard Ratio; MACE = Major adverse cardiovascular event; 95% CI: 95% confidence interval.

1. Incidence rates were defined as the total number of incident events of interest divided by the total person time at risk; 2. Event rates were defined as the total number of events, including recurrent events divided by the total person time of follow-up. Poisson exact 95% CIs will be calculated for rates
